# Antibiotic prescription practices and opinions regarding antimicrobial resistance among veterinarians in Kentucky, USA

**DOI:** 10.1371/journal.pone.0249653

**Published:** 2021-04-15

**Authors:** Agricola Odoi, Ronita Samuels, Craig N. Carter, Jackie Smith

**Affiliations:** 1 Department of Biomedical and Diagnostic Sciences, University of Tennessee, Knoxville, TN, United States of America; 2 Veterinary Diagnostic Laboratory, University of Kentucky, Lexington, KY, United States of America; University of Lincoln, UNITED KINGDOM

## Abstract

**Background:**

Inappropriate antimicrobial use (AMU) is a global concern. Opinions of veterinarians regarding AMU and its role in the development of antimicrobial resistance (AMR) may influence their prescription practices. It is important to understand these opinions, prescription practices and their potential impact on the development of AMR in order to guide efforts to curb the problem. Therefore, the objective of this study was to investigate the antimicrobial prescription practices and opinions of veterinarians in Kentucky regarding AMU and AMR.

**Methods:**

This cross-sectional study used a 30-question survey questionnaire administered to veterinarians who were members of the Kentucky Veterinary Medical Association. Survey responses from 101 participants were included in the study. Descriptive statistics were computed and associations between categorical variables assessed using Chi-square or Fisher’s exact tests. Firth logistic models were used to investigate predictors of “Compliance with prescription policies” and “Cost of antimicrobial affects prescription decisions”.

**Results:**

Almost all (93%) respondents indicated that improper AMU contributed to selection for AMR. A total of 52% of the respondents believed that antimicrobials were appropriately prescribed, while the remaining 48% believed that antimicrobials were inappropriately prescribed. Significant predictors of compliance with prescription policies were availability of prescription policy at the veterinary facility (Odds Ratio (OR) = 4.2; p<0.001) and over-prescription (OR = 0.35; p = 0.025). Similarly, significant predictors of cost of antimicrobials affecting prescription decisions were lack of post-graduate training (OR = 8.3; p = 0.008) and practice type, with large animal practices having significantly lower odds of the outcome (OR = 0.09; p = 0.004) than small animal practices.

**Conclusion:**

Most veterinarians indicated that improper AMU contributed to selection for AMR. Since the odds of compliance with prescription policies were 4-times higher among veterinarians working at facilities that had prescription policies compared to those at facilities that didn’t, more veterinary facilities should be encouraged to adopt prescription policies to help improve compliance and reduce AMR. Veterinarians would also benefit from continued professional education to help improve prescription practices, antimicrobial stewardship and curb AMR.

## Introduction

Antimicrobial resistance (AMR) has recently garnered quite a bit of attention as it has become recognized as an increasingly important global health problem with injudicious antimicrobial use (AMU) in both human and veterinary medicine being increasingly implicated as a key factor in the development of AMR [1–17]. In veterinary medicine in particular, antimicrobials are used extensively for prophylaxis, therapy and growth promotion in various animal production systems [15, 18–21]. Moreover, use of sub-lethal doses of antibiotics have been shown to lead to selection for AMR [19–22]. The contribution of AMU in companion animals to the potential development of AMR is as important as the use in production medicine. A study by Joosten *et al*, investigating AMU and AMR in companion animals, reported that broad-spectrum antimicrobials and critically important antimicrobials for human medicine represented 83% and 71% of the total number of treatments given to dogs and cats, respectively [23]. The authors suggested that addressing the issue of AMU in companion animals needed to focus on quality of use and not the quantity. They reasoned that, from a One-Health perspective, companion animals might be a source of transmission of resistance genes and/or resistant bacteria to humans [23]. An Australian study by Hur *et al* reported that antimicrobials were dispensed in 14.5% of canine consultations and that 3.8% of the consultations involved high importance rated antimicrobials [24]. Similarly, 10.8% of the cat consultations had antimicrobials dispensed, and 4.7% of the consultations involved administration of an antimicrobial of high importance rating. The investigators reported that the most common antimicrobials prescribed to cats and dogs were cefovecin and amoxycillin clavulanate, respectively [24]. With these issues in mind, it is important to understand the attitudes of veterinarians regarding AMU and the potential role of injudicious use in the development of AMR to better guide control of development of AMR.

Although guidelines for judicious use of antimicrobials have been developed [25], sometimes these guidelines are only partially implemented [26]. The World Health Organization (WHO) has strongly recommended that use of all classes of medically important antibiotics in food-producing animals be reduced and that the use of antibiotics for growth promotion and prophylaxis without appropriate diagnosis be restricted [27]. The U.S. federal government has also acted to address these concerns through the Veterinary Feed Directive (VFD) by mandating how medically important antibiotics should be administered to animals in feed and water [28]. Since there is evidence that injudicious AMU contributes to selection for resistance [19–22, 29–31], it is important to understand the prescription practices and opinions of veterinarians regarding AMR and its potential relationship with AMU practices. Although such studies have been conducted in some countries focusing on food animal veterinarians [32], there is a paucity of literature in the United States in general and Kentucky in particular. Moreover, findings from these studies are needed to better understand the prescription practices of veterinarians in not only food animal practice but also those in companion and mixed animal practices. The findings from such studies would be instrumental in guiding recommendations related to antimicrobial stewardship (AMS) to help curb the AMR problem.

Despite international, national, and local efforts to encourage AMS and to limit unnecessary AMU, the absence of universal policies to guide prescription practices limits the ability to curb development of AMR [33]. In Australia, the development of best-practice antimicrobial prescription guidelines is a key component of the animal health industry’s response to the issue of AMR [34]. These guidelines are intended to be used as decision making tools to assist with the quick selection of the most appropriate antibiotic for use in clinical settings especially when antimicrobial susceptibility data may not be available [34]. In the United States, at least 30% of antimicrobials prescribed are deemed unnecessary [35]. Therefore, it is important that clinicians are encouraged to adhere to antimicrobial prescription guidelines and policies. However, although information on prescription practices is critically important to guide this effort, there is currently paucity of information on it. Therefore, the objective of this study was to investigate antimicrobial prescription practices and opinions of veterinarians in Kentucky regarding AMU and its role in the development of AMR. This information is useful for guiding the development of effective measures to enhance compliance with policies and prescription guidelines that would be important in curbing injudicious AMU and slowing down the development of AMR.

## Methods

### Ethics approval and consent to participate

This study was approved by the Institutional Review Board (IRB) of the University of Tennessee, Knoxville (IRB approval number: UTK IRB-17-03475-XM) as exempt under 45 CFR 46 Category 2. Written informed consent was obtained from each respondent at the beginning of the online survey before the survey respondents could begin responding to the survey questions. The study used an anonymous questionnaire and did not involve animals. All data were handled in compliance with relevant guidelines.

### Study setting and design

This cross-sectional study used a 30-item survey questionnaire designed to collect data on opinions, attitudes and antimicrobial prescription practices of veterinarians in Kentucky (USA). The survey instrument was adopted from two previous survey questionnaires [36, 37] and included questions on antimicrobial prescription practices, attitudes on AMU and how it relates to the development of AMR. The original questionnaires were improved by adding questions addressing factors thought to be potentially associated with prescription practices, and opinions regarding the role of AMU in the development of AMR.

The questionnaire was designed to take 20–30 minutes to complete and was divided into 6 sections: Demographic Information, Veterinary Education, Antimicrobial Prescription Practices, Factors Associated with Prescribing Habits, Opinions About Prescription Practices, and Opinions About AMR. These six sections contained both open-ended and close-ended questions consisting of a combination of yes/no questions, multiple choice questions as well as 5-point Likert scale questions (1-strongly agree; 2-agree; 3-neither agree nor disagree; 4-disagree; 5-strongly disagree). Pretest of the questionnaire was done in two steps: (a) The first step involved evaluation of the questionnaire by experts in the field from the University of Tennessee, College of Veterinary Medicine. This helped identify all important issues to be addressed in the questionnaire as well as identify potentially ambiguous questions. (b) The 2^nd^ step of the pretest involved administration of the questionnaire to a small sample of 10 respondents from the intended study population. This helped identify confusing, ambiguous, or misleading questions. For multiple choice questions, this also allowed for the inclusion of additional response categories identified during this step of the pretest.

### Survey administration

The survey questionnaire was uploaded to Qualtrics [38] for online access. Veterinarians who were members of the Kentucky Veterinary Medical Association (KVMA) were contacted by email (through the association leadership) and requested to anonymously participate in the study via a non-personalized Web link provided in the email.

The initial email, with a link to the online questionnaire survey, was sent to the KVMA list-serve in April 2017. Due to association policy, investigators did not have access to the exact number of members reached by email and hence the potential number of participants could not be attained. Therefore, it was not possible to estimate the response rate. However, to increase response rate, a total of 6 reminder emails were sent to the list-serve between May and October 2017 requesting list-serve members to complete the survey and thanking those that had already completed. Regarding sample size consideration, assuming the proportion of respondents that over-prescribe antibiotics is 0.52, a 95% confidence level and an allowable error of 0.1, the required sample size was estimated at 99 respondents.

### Data analysis

All statistical analyses were performed in SAS 9.4 [39]. The distribution of demographic variables and their 95% confidence intervals were computed. The demographic variables considered for analysis were: sex of participant, city, veterinary practice, veterinary facility, length of time at facility, number of veterinarians at the facility, hours worked per week, and year of graduation. Veterinary practice was classified as small animal, large animal or mixed practice. A small animal practice was defined as one that primarily provides veterinary care to a variety of companion small animal species such as dogs, cats, rabbits, mice, rats, ferrets, guinea pigs and other pets. Large animal practice, on the other hand, was defined as one that provides care primarily to farm animals such as cattle, horses, pigs, goats, sheep, llamas and alpacas. Finally, a mixed animal practice was defined as one that provides veterinary care to both farm and companion small animals.

Most of the open-ended questions involved numeric entries such as years of experience, number of veterinarians in the practice, hours of work per week, etc. Continuous variables were assessed for normality of distribution using Shapiro-Wilks test (α = 0.05). Medians and interquartile ranges were computed for non-normally distributed continuous variables, otherwise means and standard errors were used. Frequency distributions, percentages and 95% confidence intervals of the categorical responses to the survey questions were also computed. Due to the small number of responses in some of the response categories, the 5-point Likert Scale variables, “Improper use of antimicrobials contributes to selection for AMR” and “My colleagues over prescribe antimicrobials” were recoded to 1-strongly agree or agree, 2-neither agree nor disagree, 3-disagree or strongly disagree.

Univariable associations of opinion and prescription practice variables were assessed using Chi-square or Fisher’s exact test as appropriate, using a relaxed alpha of 15% (α = 0.15) for these initial assessments. Predictors of “Compliance with antimicrobial prescription policies” (Yes/No) and “Cost Affects Prescription Decisions” (Yes/No) were investigated using Firth logistic regression models. All potential predictor variables that had univariable associations with the above two outcome variables at an alpha-level of 0.15 (based on Chi-square or Fisher’s exact tests) were considered for inclusion in the multivariable Firth logistic models which were built using backwards elimination procedure. However, for the multivariable Firth models, statistical significance was assessed using an alpha of 0.05. Firth logistic models were chosen for these data because the maximum likelihood estimation of the ordinary logistic regression suffers from small-sample bias. Therefore, the Firth logistic models provide better estimates than the ordinary logistic models for these data [40]. Confounding was assessed by examining whether the removal of a variable from the model resulted in a change of >20% in the coefficients of any of the other variables already in the model. Hosmer-Lemeshow test was used to assess goodness-of-fit of the final models.

## Results

### Respondent profile

A total of 101 veterinarians participated in the study, 57.4% of whom were female and 42.6% were male (Table 1). Throughout the results, the effective sample size varies across questions (variables) because none of the questions in the questionnaire were mandatory and therefore some respondents did not answer some of the questions. Just under a half (44.9%) of the respondents were from two cities, Lexington (26.5%) and Louisville (18.4%). Most of the respondents worked in small animal (58.0%) and mixed animal practices (23.0%). More than half of the veterinarians worked at primary care facilities (55.0%), while 29.0% worked at veterinary hospitals and 16.0% were in referral hospitals (Table 1). The median number of years of experience of the respondents was 12 years (interquartile range: 3, 27). The majority (84.5%) of the respondents worked in facilities that had ≤10 veterinarians on payroll (Table 1). Although the median number of veterinarians per veterinary facility was 3 (interquartile range: 1; 7), the number of veterinarians per facility ranged from 1 to 65 with 30% of the facilities having 1 veterinarian and 1 facility having 65 veterinarians. The median number of years the respondents had worked at their practice was 11.5 years (interquartile range: 3, 27), but ranged from <1 year to 50 years.

**Table 1:** Respondent demographic profile from a survey of veterinarians in Kentucky, 2017.

Variable	Number	Percentage	95% CI^1^
**Sex (Single-answer Question)**	**n = 101**		
Female	58	57.4	47.2, 67.2
Male	43	42.6	32.8, 52.8
**City (Free Text)**	**n = 98**		
Lexington	26	26.5	18.1, 36.4
Louisville	18	18.4	11.3, 27.5
Other	54	55.1	44.7, 65.2
**Veterinary Practice (Single-answer Question)**	**n = 100**		
Large Animal	19	19.0	11.8, 28.1
Mixed	23	23.0	15.2, 32.5
Small Animal	58	58.0	47.7, 67.8
**Veterinary Facility (Single-answer Question)**	**n = 100**		
Primary Care	55	55.0	44.7, 65.0
Referral Hospital	16	16.0	9.4, 24.7
Veterinary Hospital	29	29.0	20.4, 38.9
**Veterinarians per Facility (Derived Variable)**	**n = 97**		
≤10	82	84.5	75.8, 91.1
>10	15	15.5	8.9, 24.2

^1^95% Confidence Interval.

### Training on antibiotics during veterinary education

About half (49.5%) of the veterinarians indicated that antibiotics were emphasized in multiple courses during the pre-clinical years of their veterinary education (Table 2). However, the number of respondents indicating that antibiotics were emphasized in multiple courses during the clinical years rose to 67.0% (Table 2). Pharmacologist/clinical pharmacologists were mostly responsible (34.8%) for the training on antibiotics, followed by clinicians (27.4%) and clinical microbiologists (18.3%) (Table 2). A total of 25.8% of the respondents had post-graduate education (Table 2).

**Table 2:** Responses of veterinarians in Kentucky regarding their training on antibiotics, 2017.

Question/Response	Number	Percentage	95% CI^1^
**What was the emphasis on antibiotics in veterinary school education (non-clinical years)?**	**n = 97**		
**(Single-answer Question)**			
Topic was not covered	1	1.0	0.02, 6.0
Light emphasis	21	21.7	13.9, 31.2
Covered thoroughly in one course	27	27.8	19.2, 37.9
Emphasized in multiple courses	48	49.5	39.2, 59.8
**What was the emphasis on antibiotics in your veterinary school education (clinical years)?**	**n = 97**		
**(Single-answer Question)**			
Topic was not covered	1	1.0	0.02, 6.0
Light emphasis	26	26.8	18.3, 36.8
Covered thoroughly in one course	5	5.2	1.7, 11.6
Emphasized in multiple courses	65	67.0	56.7, 76.2
**What was the background of the person primarily responsible for your education on antibiotics during your veterinary education?**^2^	**n = 101**		
**(Select All that Apply)**			
Clinical pharmacist	20	12.2	7.6, 18.2
Clinical microbiologist	30	18.3	12.7, 25.1
Clinician	45	27.4	20.8, 34.9
Pharmacologist/clinical pharmacologist	57	34.8	27.5, 42.6
Toxicologist	7	4.3	1.7, 8.6
Don’t know his/her background	5	3.1	1.0, 7.0
**Do you hold any additional post-graduate qualifications? (Single-answer Question)**	**n = 97**		
Yes	25	25.8	17.43, 35.65
No	72	74.2	64.35, 82.58

^1^95% Confidence Interval.

^2^The percentages total to >100% because some respondents selected more than one response category.

### Antimicrobial prescription practices

Just over a quarter (26.4%) of the veterinarians received information regarding antimicrobials from textbooks/drug handbooks and continuing professional development courses (26.0%) (Table 3). Peer reviewed scientific literature (18.4%) and pharmaceutical companies (15.6%) were also cited as sources of information on antimicrobials (Table 3). A total of 4.9% of the veterinarians received information regarding antimicrobials and their usage from their practice’s policies (Table 3).

**Table 3:** Antimicrobial use and prescription practices among veterinarians in Kentucky, 2017.

Question/Responses	Number	Percentage	95% CI
**What are the main sources that you use to receive current information on antimicrobials and their use?**	**n = 93**		
**(Select All that Apply)**			
Practice policy	14	4.9	2.7, 8.0
Pharmaceutical companies	45	15.6	11.6, 20.3
Veterinary medicine directorates	17	5.9	3.5, 9.3
Peer reviewed scientific literature	53	18.4	14.1, 23.4
Textbook/drug handbook	76	26.4	21.4, 31.9
Continuing professional development courses	75	26.0	21.1, 31.5
Other	8	2.8	1.2, 5.4
**Can you prescribe antibiotics without supervision, approval, or additional oversight? (Single-answer Question)**	**n = 93**		
Yes	86	92.5	85.1, 96.9
No	7	7.5	3.1, 14.9
**Does your veterinary facility or practice have a policy concerning antibiotic prescription? (Single-answer Question)**	**n = 92**		
Yes	39	42.4	32.2, 53.1
No	53	57.6	46.9, 67.9
**On Average, how often do you prescribe antibiotics?**	**n = 93**		
**(Single-answer Question)**			
Multiple times per day	71	76.3	66.4, 84.5
Once per day	4	4.3	1.2, 10.7
Once every two days	4	4.3	1.2, 10.7
Once per week	6	6.5	2.4, 13.5
Once every two weeks	1	1.1	0.0, 5.9
Once per month	1	1.1	0.0, 5.9
Once every two to four months	4	4.3	1.2, 10.7
Quarterly	0	0.0	0.0, 0.0
Biannually	2	2.2	0.3, 7.6
Annually	0	0.0	0.0, 0.0
**Is there any antibiotic that you do not feel comfortable prescribing? (Single-answer Question)**	**n = 93**		
Yes	50	53.8	43.1, 64.2
No	43	46.2	35.8, 56.9
**Do any of the factors below affect your decision when choosing to prescribe an antibiotic to a patient?**	**n = 93**		
**(Select All that Apply)**			
Cost of antibiotic	82	24.6	20.0, 29.5
Client insurance	3	0.9	0.2, 2.6
Client expectations	28	8.4	65.5, 93.2
Route of administration	89	26.7	22.0, 31.7
Frequency of patient visits	28	8.4	5.6, 11.9
Risk of potential adverse drug reaction	78	23.4	18.9, 28.3
Other	26	7.8	5.2, 11.2
**You always rely on clinical signs and symptoms when prescribing an antibiotic (Single-answer Question).**	**n = 92**		
Strongly agree	41	44.6	34.2, 55.3
Agree	38	41.3	31.1, 52.1
Neither agree nor disagree	10	10.9	5.3, 19.1
Disagree	3	3.3	0.7, 9.3
Strongly disagree	0	0.00	0.0, 0.0
**You rely on laboratory results before prescribing an antibiotic (Single-answer Question).**	**n = 92**		
Strongly agree	16	17.4	10.3, 26.7
Agree	35	38.0	28.1, 48.8
Neither agree nor disagree	29	31.5	22.2, 42.0
Disagree	8	8.7	3.8, 16.4
Strongly disagree	4	4.4	1.2, 10.8
**What are your feelings concerning antibiotic prescription at your facility or practice? (Single-answer Question)**	**n = 91**		
All antibiotics are under-prescribed	1	1.1	0.0, 6.0
Some antibiotics are under-prescribed	6	6.6	2.5, 13.8
All antibiotics are appropriately prescribed	47	51.7	40.9, 62.3
Some antibiotics are over-prescribed	34	37.4	27.4, 48.1
All antibiotics are over-prescribed	3	3.3	0.7, 9.3
**Do you feel like you sometimes over-prescribe antibiotics?**	**n = 92**		
**(Single-answer Question)**			
Yes	42	45.7	35.2, 56.4
No	50	54.4	43.6, 64.8
**Your colleagues over-prescribe antibiotics (Single-answer Question)**	**n = 92**		
Strongly agree	6	6.5	2.4, 13.7
Agree	34	37.0	27.2, 47.7
Neither agree nor disagree	39	42.4	32.2, 53.1
Disagree	13	14.1	7.7, 23.0
Strongly disagree	0	0.0	0.0, 0.0
**Veterinarians at your practice or facility always comply with antibiotic prescription policies (Single-answer Question).**	**n = 92**		
Strongly agree	20	21.7	13.8, 31.6
Agree	35	38.0	28.1, 48.8
Neither agree nor disagree	32	34.8	25.2, 45.4
Disagree	4	4.4	1.2, 10.8
Strongly disagree	1	1.1	0.0, 5.9

^1^95% Confidence Interval.

However, more than half (57.6%) of the respondents did not have policies concerning antimicrobial prescription at their practices (Table 3). Almost all (92.5%) the veterinarians indicated that they were able to prescribe antimicrobials without supervision, or oversight. Although more than half of the practices did not have antimicrobial prescription policies, 76.3% of the respondents reported prescribing antimicrobials multiple times a day (Table 3). Moreover, more than half (53.8%) of the respondents reported that they were not comfortable prescribing at least one type of antimicrobial (Table 3). Of these (n = 50), 20% (10/50) were not comfortable prescribing chloramphenicol. Other antimicrobials that these veterinarians were uncomfortable prescribing were Trimethoprim/Sulfadiazine (TMS) or Sulfamethoxazole (10%), aminoglycosides (8%), vancomycin (6%), fluoroquinolones (6%), and gentamycin (4%). The rest of the respondents did not specify the antimicrobial they were not comfortable prescribing.

### Factors influencing antimicrobial prescription practices

The veterinarians reported that their decisions to prescribe antimicrobials were based on route of administration (26.7%), cost (24.6%) and risk of potential adverse reactions (23.4%) (Table 3). The majority of veterinarians either strongly agreed (44.6%) or agreed (41.3%) that they always relied on clinical signs and symptoms to prescribe antimicrobials. However, slightly more than half, (17.4% strongly agreed and 38.0% agreed) relied on laboratory results before prescribing antimicrobials (Table 3).

Consideration of cost of antibiotics in prescription decisions was significantly (p = 0.0057) associated with type of veterinary practice. The percentage of large animal veterinarians (66.7%; 12/18) who considered cost in prescription decisions was significantly lower than those in both small animal practice (94%; 52/55; p = 0.0013) and mixed animal practice (95%; 19/20; p = 0.0123). However, the percentage of veterinarians that considered cost was not significantly different (p = 0.869) between small animal and mixed animal practices.

Cost of antibiotic being considered in prescription decisions was also significantly (p<0.001) associated with veterinary facilities. The percentages of primary care facility veterinarians (98.0%; 48/49) that considered cost in prescription decisions was significantly (p = 0.0485) larger than that of veterinarians in veterinary hospitals (89.3%; 25/28). Additionally, the percentage of referral facility veterinarians (62.5%; 10/16) that considered cost in prescription decisions was significantly lower than those working at both primary care facilities (p<0.001) and veterinary hospitals (p = 0.017).

Significantly (p = 0.0026) more veterinarians with post-graduate training (34.8%; 8/23) than those without post-graduate training (7.1%; 7/70) indicated that efficacy of antibiotics affected their prescription decisions. By contrast, significantly (p = 0.0133) more veterinarians without post-graduate training (94.3%; 66/70) than those with post-graduate training (73.9%; 17/23) indicated that cost of antibiotics affected their decision on choice of antibiotics.

Consideration of client insurance when making prescription decisions significantly (p = 0.0063) differed across practice types with 16.7% (3/18) of large animal veterinarians and 0% of either small or mixed animal veterinarians considering this factor. There was also a significant association between the type of veterinary practice and consideration of the fact that the “client expects antibiotics”. The percentage of large animal veterinarians (61.1%; 11/18) who considered this factor during their prescription was significantly higher than both those in small animal practice (21.8%; 12/55; p = 0.009) and mixed animal practice (25%; 5/20; p = 0.0122). However, there were no significant differences (p = 0.770) in the percentages of veterinarians that considered this factor in small animal and mixed animal practices.

### Opinions on antimicrobial prescription practices

Approximately half (51.7%) of the veterinarians believed that antimicrobials were appropriately prescribed at their practice, while 37.4% believed that some antimicrobials were over-prescribed (Table 3). Slightly more than half of the veterinarians in this study did not believe that they ever over-prescribed antimicrobials, although 45.7% believed that they did indeed over-prescribe antimicrobials (Table 3). A total of 43.5% either strongly agreed (6.5%) or agreed (37.0%) that their colleagues over-prescribed antimicrobials (Table 3). Additionally, 59.7% (21.7% strongly agreed and 38.0% agreed) believed that their colleagues always complied with antimicrobial prescription policies (Table 3). However, 24% (3.3% strongly agree and 20.7% agreed) believed that antimicrobial prescription policies actually contributed to a change in the incidence of AMR at their practice (Table 4).

**Table 4:** Opinions, of veterinarians in Kentucky, on the role of antimicrobial use on development of antimicrobial resistance, 2017.

Question/Response	Number	Percentage	95% CI^1^
**Antibiotic prescription policies are contributing to a change in the frequency of antimicrobial resistance at your facility or practice (Single-answer Question).**	**n = 92**		
Strongly agree	3	3.3	0.7, 9.2
Agree	19	20.7	12.9, 30.4
Neither agree nor disagree	49	53.3	42.6, 63.7
Disagree	14	15.2	8.6, 24.2
Strongly disagree	7	7.6	3.1, 15.1
**Improper use of antibiotics contributes to selection for antimicrobial resistance (Single-answer Question).**	**n = 87**		
Strongly agree	44	50.6	39.6, 61.5
Agree	37	42.5	32.0, 53.6
Neither agree nor disagree	6	6.9	2.6, 14.4
Disagree	0	0.0	0.0, 0.0
Strongly disagree	0	0.0	0.0, 0.0
**How does improper use of antibiotics affect selection for antimicrobial resistance? (Single-answer Question)**	**n = 81**		
It does not affect selection for AMR^2^	10	12.35	6.1, 21.5
Improper use of antibiotics affects selection for AMR	71	87.65	78.5, 93.9
**Improper prescribing habits among your colleagues is affecting the selection for antibiotic resistance in your facility (Single-answer Question).**	**n = 86**		
Strongly agree	1	1.2	0.0, 6.3
Agree	16	18.6	11.0, 28.5
Neither agree nor disagree	42	48.8	37.9, 59.9
Disagree	21	24.4	15.8, 34.9
Strongly disagree	6	7.0	2.6, 14.6
**There has been an increase in the number of cases of antimicrobial resistance at your facility or practice.**	**n = 86**		
**(Single-answer Question)**			
Strongly agree	1	1.2	0.0, 6.3
Agree	12	14.0	7.4, 23.1
Neither agree nor disagree	30	34.9	24.9, 45.9
Disagree	33	38.4	28.1, 49.5
Strongly disagree	10	11.6	5.7, 20.4

^1^95% Confidence Interval.

^2^Antimicrobial resistance.

There was a significant (p = 0.0051) association between a practice having antimicrobial prescription policy and respondent’s opinion regarding whether veterinarians in their practice always complied with antimicrobial prescription policies. More respondents (76.9%; 30/39) from practices with antimicrobial prescription policies agreed (35.9% strongly agree; 41% agree) that veterinarians in their practice always complied with prescription policies compared to 46.1% (24/52) (11.5% strongly agree; 34.6% agree) from practices without antimicrobial prescription policies. Significantly (p = 0.047) fewer veterinarians with post-graduate training (27.3%; 6/22) indicated that they sometimes over-prescribed antibiotics compared to veterinarians without post-graduate training (51.4%; 36/70).

### Opinions on AMR

Almost all respondents (93%) agreed that improper use of antimicrobials contributed to selection for AMR. Nearly 20% either strongly agreed (1.2%) or agreed (18.6%) that improper antimicrobial prescription practices among their colleagues were affecting the selection for AMR at their facility (Table 4). However, 15.2% (1.2% strongly agree; 14.0% agree) of the veterinarians thought that there had been an increase in the incidence of AMR at their practice (Table 4).

### Predictors of “compliance with antimicrobial prescription policies” and “cost affects prescription decisions”

Significant predictors of “compliance with antimicrobial prescription policies” were “the practice having antibiotic prescription policy” (p<0.001) and “over prescription of antimicrobials by the respondents” (p = 0.025). The odds of “compliance with antimicrobial prescription policies” was 4.2 times higher among respondents whose practice had antimicrobial prescription policies compared to respondents whose practice did not have prescription policies (Table 5). Additionally, the odds of “compliance with antimicrobial prescription policies” tended to be lower (Odds Ratio (OR) = 0.35) among respondents who said they felt they “sometimes over-prescribe antimicrobials” compared to those who did not (Table 5). The Hosmer-Lemeshow goodness-of-fit test for the model indicated no evidence of lack of fit (p = 0.733).

**Table 5:** Multivariable logistic model results showing predictors “compliance with antimicrobial prescription policies” and “cost of antimicrobial affects prescription decisions” among veterinarians in Kentucky, 2017.

Outcome/Model	Predictor	Adjusted Odds Ratio	95% CI^1^	p-value
**Compliance with antimicrobial prescription policies**				
	***Practice has antibiotic prescription Policy***			***0*.*003***
	Yes	4.21	1.61, 11.0	0.003
	No			
	***Over prescription by Respondent***			***0*.*025***
	Yes	0.35	0.14, 0.87	0.025
	No	referent		
**Cost Affects Prescription Decisions**				
	***Post-graduate training***			***0*.*008***
	No	8.28	1.74, 39.48	0.008
	Yes	Referent		
	***Practice***			***0*.*011***
	Large Animal	0.09	0.02, 0.47	0.004
	Mixed Animal	0.88	0.11, 7.13	0.276
	Small Animal	referent		

^1^95% Confidence Interval.

The significant predictors of “Cost of antibiotic affects prescription decision” were post-graduate training (p = 0.008) and veterinary practice (p = 0.011). The odds of cost of antibiotic affecting prescription decision was 8.3 times higher among veterinarians who did not have post-graduate training compared to those that had post-graduate training. Furthermore, the odds of cost of antibiotic affecting prescription decision was lower among large animal veterinarians (OR = 0.09; p = 0.004) compared to small animal veterinarians. However, there were no differences in the odds between small animal and mixed animal veterinarians (OR = 0.88; p = 0.276). Again, the Hosmer-Lemeshow goodness-of-fit test for this model indicated no evidence of lack of fit (p = 0.960).

## Discussion

This study used a survey questionnaire to investigate antimicrobial prescription practices and opinions of Kentucky veterinarians regarding development of AMR. Nearly half of the veterinarians in this study indicated that antimicrobials were emphasized in multiple courses during their pre-clinical years of veterinary education, but this number rose to 67% during the clinical years which indicates an increase in antimicrobial training focus as veterinary students progressed through their curriculum. Although many studies worldwide have focused on the knowledge and perceptions of medical and pharmacy students regarding antimicrobials and AMR [41–48], only a small number of studies have focused on the number of courses that cover antimicrobials in veterinary student training [49, 50] or examined the breadth of coverage concerning antimicrobials in both pre-clinical and clinical years of study [43, 50]. Thus, there is currently a paucity of information on the depth of education on antimicrobials during the pre-clinical and clinical years of veterinary education. Although, a nationwide study in the U.K. by Castro-Sanchez found that AMS is included in the majority of veterinary medicine courses in the U.K. [51], it is obvious that more similar studies need to be conducted in other parts of the world to assess both veterinarians and veterinary students to better understand attitudes and practices related to AMU so as to better inform efforts to improve AMS and curb AMR.

A Nigerian study investigated the perceptions, attitudes and knowledge of final year veterinary students and reported that 17% had heard about AMS and only 7% knew the meaning of AMS [49]. Moreover, only 17% of the respondents felt that their education on AMR was adequate for their veterinary career. However, most of the them believed that strong knowledge of antimicrobials is important for their future veterinary career and would like more education on AMR and AMS [49]. The study concluded that Nigerian veterinary students’ perception and knowledge of AMR and AMS were poor and therefore creates doubt about their preparedness to practice AMS. A similar South African study of medical students reported that 95% of the students reported that they would appreciate more education on appropriate use of antibiotics and only 33% felt confident to prescribe antibiotics [51]. This may indicate that the identified gaps in the training on AMS may not be limited to veterinary professionals. Indeed, an Australian study investigating the knowledge and perceptions of veterinary students regarding AMS and biosecurity reported that only 34% thought pharmacology teaching was adequate and only 20% said that teaching in lectures matched clinical teaching [50]. The authors recommended that efforts need to be made to harmonize pre-clinical and clinical teaching, and that greater emphasis is needed on AMS [50]. Although the above examples point to a few countries, it is possible that the situation observed may not be unique to these countries. This calls for more similar studies in more countries to identify the gaps in training of both veterinary and medical professionals regarding AMS. It is through identifying these gaps and improving the curricular that we will better be able to improve AMS and AMU among both medical and veterinary professionals of tomorrow who will potentially have meaningful impacts on the global AMR problem.

### Antimicrobial prescription practices

The majority of veterinarians received information regarding antimicrobials and their use from textbooks/drug handbooks and continuing professional development courses and only 5% received this information from their practice’s policies. This differed from the findings of a study in the U.K. by Coyne *et*. *al*., that reported that veterinarians relied on their own experience and colleagues as well as the history of the farm [52]. However, mixed species practitioners consulted a wider variety of information sources on antimicrobials and were more likely to seek information from colleagues compared with practitioners working within specialist pig practices [52]. It is important that veterinarians make evidence-based clinical decisions based on information from reputable sources to better guide decisions on AMU and AMS so as to curb the AMR problem [53]. This highlights the critical need for AMU policies in veterinary practices. The potential benefit of having veterinary practices adopt antimicrobial prescription policies is evidenced by the findings of the current study that revealed that the odds of compliance with antimicrobial prescription policies was 4-times higher among veterinarians whose veterinary practices had prescription policies compared to those whose practices did not. Moreover, lack of compliance with prescription policies was found to be associated with over-prescription. Therefore, more veterinary practices need to be encouraged to adopt AMU policies to better guide their veterinarians and help improve AMS and reduce AMR.

Almost all the veterinarians in the current study were able to prescribe antimicrobials without oversight. However, more than half indicated that their practice did not have a policy concerning antimicrobial prescription. This is similar to the findings of an Australian study by Hardefeldt et. al., that found that veterinary practices rarely had antimicrobial prescription policies [54]. Although no universal guideline or policy exists for antimicrobial prescription in veterinary medicine, the American Veterinary Medical Association (AVMA) House of Delegates voted in 2018 to enact a policy on AMS [55]. One of the objectives of the vote was to improve veterinary antimicrobial prescription practices and encourage collaborations between veterinary and human medical professionals. Such initiatives are critically important in the fight against AMR. Based on the findings of this study, it was encouraging to see that compliance with antimicrobial prescription policies was higher among veterinarians working in facilities with prescription policies. This implies that as more veterinary facilities adopt prescription policies, more veterinarians will likely be compliant with AMU policies which would potentially result in overall improvement of AMS and potential reduction in AMR.

That more than 50% of the respondents were uncomfortable prescribing some antimicrobials is not uncommon and is consistent with the findings from a U.S. study by Jacob *et*. *al*., investigating opinions of clinical veterinarians at a U.S. veterinary teaching hospital [36]. Jacob and co-workers reported that 46% of survey respondents felt uncomfortable prescribing at least one class of antimicrobials [36]. This issue could be addressed through regular continuing professional education and adopting antimicrobial prescription policies by more veterinary practices. These would help improve AMS competencies and ensure that all veterinarians adhere to AMU and AMS policies. The fact that more respondents (76.9%) from veterinary facilities with antimicrobial prescription policies agreed that veterinarians in their facilities always complied with prescription policies compared to facilities without antimicrobial prescription policies is an indication of the potential long-term impact of having more facilities adopt these policies.

### Antimicrobial prescription practices and AMR

Route of administration, cost of antimicrobial and risk of potential adverse reactions were the three most common factors reported to guide veterinarians’ decision to prescribe antimicrobials. A qualitative study by Mateus *et*. *al*., in the U.K., investigated factors associated with AMU in small animal veterinary practices and found that antimicrobial prescription was influenced not only by veterinarian’s preference for certain substances and previous experience, but also by perceived efficacy, ease of administration of formulations, perceived compliance, willingness of pet owners to give the antimicrobial as well as animal characteristics [56]. It is worth pointing out that the study by Mateus *et*. *al*. identified cost as a factor only in low socioeconomic areas or areas of varying socioeconomic status [56]. In the current study, the fact that the odds of cost affecting prescription decisions was 8-times higher among veterinarians who did not have post-graduate training compared to those that had post-graduate training is an indication of the general importance of veterinary education and continuing professional education of veterinarians to improve AMU practices. This is further supported by the fact that significantly more veterinarians with post-graduate training considered efficacy of antibiotics to guide their prescription decisions than those without post-graduate training. Moreover, significantly fewer veterinarians with post-graduate education indicated that they over-prescribed antibiotics compared to their counter-parts without post-graduate training. Additionally, significantly more veterinarians without post-graduate training considered cost in prescription decisions compared to their counter-parts that had post-graduate training. These findings seem to suggest that more training and use of prescription policies enhance the ability of veterinarians to consider more clinically relevant factors to help make more prudent prescription decisions. Therefore, it seems that more professional education and adopting prescription policies by more facilities would go a long way in improving AMS and addressing the AMR problem.

Over 80% of the veterinarians in the current study either strongly agreed or agreed that they always relied on clinical signs and symptoms before prescribing antimicrobials. However, only slightly more than half, either strongly agreed or agreed that they relied on laboratory results before prescribing antimicrobials. Empirical AMU is not uncommon especially in rural areas that have limited access to laboratory facilities. A U.S. study by Fowler *et*. *al*., found that only 36% of veterinarians reported ordering culture and sensitivity testing ‘often’ or ‘always’ when treating presumptive bacterial infections [57]. An Italian study by Barbarossa *et*. *al*., also identified low usage of laboratory testing with only 7% of the respondents routinely waiting for laboratory results before starting treatment [58]. The fact that more than half of the veterinarians in our study reported relying on laboratory testing may be due to relatively good access to laboratory testing in the two large cities in Kentucky from where a significant proportion of respondents practiced.

About 46% of the veterinarians in the current study felt that they sometimes over-prescribed antimicrobials. This is comparable to findings by Ekakoro and Okafor in Tennessee that reported that 52% of the respondents believed antimicrobials are being over-prescribed [59] but much lower than findings by Jacob *et*. *al*. who reported that antimicrobial over-prescription was identified by 88% of respondents in their U.S. based study [37].

The majority (93%) of the veterinarians in this study indicated that improper use of antimicrobials contributed to selection for AMR. This is much higher than reports by an Australian study which reported that over 50% of the respondents indicated that veterinary AMU had a moderate contribution to the overall AMR problem [54]. An Italian study by Pozza *et*. *al*. reported that 85.8% of cattle and 69.8% of pig veterinarians agreed with the statement ‘the preventive use of antibiotics fosters the development of AMR’ and that 64.5% of cattle and 69.1% of pig veterinarians indicated that they suggest/prescribe alternative approaches to the use of antimicrobials [32]. The study by Pozza *et*. *al*. also reported that 69.4% of cattle veterinarians and 59.4% of pig veterinarians somewhat or strongly agreed that use of broad-spectrum antibiotics in farm animals increases AMR [32]. They also reported that the factors that affected veterinarians’ decisions on the choice of antibiotics ranged from efficacy, training/scientific knowledge, and field experience, to duration of the withdrawal period. The authors report that the following factors had less impact on veterinarians’ decisions on the choice of antibiotics: opinion of the farmer, pharmaceutical representatives, and advertisement [32]. All these point to the need for continued prudent clinical decision-making based on sound knowledge. Thus, continuing professional education and use of prescription policies by all veterinary facilities cannot be over-emphasized.

### Strengths and limitations

The study provides useful information on antimicrobial prescription practices and opinions on how AMU affects development of AMR. This information is useful for guiding efforts aimed at development of AMS programs and in education of veterinarians on prudent AMU in order to slow down the development of AMR. An analytical strength of the study is the fact that it used Firth logistic models which have the benefit of using penalized likelihood to reduce small-sample bias associated with maximum likelihood estimation [60]. Use of penalized likelihood in logistic models also has the advantage of producing finite, consistent estimates of regression parameters in situations where maximum likelihood estimates do not exist due to complete or quasi-complete separation [61, 62]. However, this study is not without limitations. Since the investigators did not have access to the mailing list or information on the number of association members who received the request to participate in the study, it was not possible to compute the study response rate. Additionally, the Web link sent to potential respondents was not personalized (in order to maintain anonymity) and, therefore, it was not possible to track if any participants completed the survey multiple times.

## Conclusions

This study provides useful information on prescription practices, perceptions, and attitudes of veterinarians regarding AMU, AMS and role of AMU on the development of AMR. Most veterinarians indicated that improper AMU contributed to selection for AMR. Since the odds of compliance with antimicrobial prescription policies was 4-times higher among veterinarians working in facilities with prescription policies compared to those that didn’t, veterinary facilities should be encouraged to adopt use of prescription policies to improve compliance and AMU with the goal of reducing AMR. Veterinarians would also benefit from continued professional education to help improve prescription practices, AMS and curb AMR.

## Supporting information

S1 File The questionnaire survey raw data, in Microsoft Excel format, generated during the study. (XLSX)

## Abbreviations

AMR: Antimicrobial resistance
AMS: Antimicrobial Stewardship
AMU: Antimicrobial Use
KVMA: Kentucky Veterinary Medical Association
WHO: World Health Organization
VFD: Veterinary Feed Directive
FDA: U.S. Food and Drug Administration
CDC: Centers for Disease Control and Prevention
AVMA: American Veterinary Medical Association
UKVDL: University of Kentucky Veterinary Diagnostic Laboratory
US: United States
U.K.: United Kingdom
